# Heavy menstrual bleeding among women aged 18–50 years living in Beijing, China: prevalence, risk factors, and impact on daily life

**DOI:** 10.1186/s12905-019-0726-1

**Published:** 2019-02-04

**Authors:** Chengyi Ding, Jing Wang, Yu Cao, Yuting Pan, Xueqin Lu, Weiwei Wang, Lin Zhuo, Qinjie Tian, Siyan Zhan

**Affiliations:** 1 Research Department of Epidemiology and Public Health, University College London, London, UK; 2 Murdoch Children’s Research Institute, Royal Children’s Hospital, Parkville, VIC 3052 Australia; 3 Department of Epidemiology and Biostatistics, School of Public Health, Peking University, Beijing, China; 4 The National Clinical Research Center for Mental Disorders & Beijing Key Laboratory of Mental Disorders, Beijing Anding Hospital, Capital Medical University, Beijing, China; 5 Department of Obstetrics and Gynecology, Peking Union Medical College Hospital, Chinese Academy of Medical Sciences, Peking Union Medical College, Beijing, China

**Keywords:** China, Cross-sectional study, Heavy menstrual bleeding, Prevalence, Quality of life, Risk factors

## Abstract

**Background:**

Heavy menstrual bleeding (HMB) has been shown to have a profound negative impact on women’s quality of life and lead to increases in health care costs; however, data on HMB among Chinese population is still rather limited. The present study therefore aimed to determine the current prevalence and risk factors of subjectively experienced HMB in a community sample of Chinese reproductive-age women, and to evaluate its effect on daily life.

**Methods:**

We conducted a questionnaire survey in 2356 women aged 18–50 years living in Beijing, China, from October 2014–July 2015. A multivariate logistic regression model was used to identify risk factors for HMB.

**Results:**

Overall, 429 women experienced HMB, giving a prevalence of 18.2%. Risk factors associated with HMB included uterine fibroids (adjusted odds ratio [OR] =2.12, 95% confidence interval [CI] = 1.42–3.16, *P* < 0.001) and multiple abortions (≥3) (adjusted OR = 3.44, 95% CI = 1.82–6.49, *P* < 0.001). Moreover, women in the younger age groups (≤24 and 25–29 years) showed higher risks for HMB, and those who drink regularly were more likely to report heavy periods compared with never drinkers (adjusted OR = 2.78, 95% CI = 1.20–6.46, *P* = 0.017). In general, women experiencing HMB felt more practical discomforts and limited life activities while only 81 (18.9%) of them had sought health care for their heavy bleeding.

**Conclusions:**

HMB was highly prevalent among Chinese women and those reporting heavy periods suffered from greater menstrual interference with daily lives. More information and health education programs are urgently needed to raise awareness of the consequences of HMB, encourage women to seek medical assistance and thus improve their quality of life.

**Electronic supplementary material:**

The online version of this article (10.1186/s12905-019-0726-1) contains supplementary material, which is available to authorized users.

## Background

Heavy menstrual bleeding (HMB) is defined as excessive menstrual blood loss which interferes with a woman’s physical, social, emotional, and/or material quality of life. It can occur alone or in combination with other symptoms [1]. The objective definition of a total menstrual blood loss (MBL) exceeding 80 mL per menstruation is now only used in studies involving physiological mechanisms because the amount of blood loss does not always correlate with women’s perception of their MBL or the impact of bleeding on their lives [2, 3].

About 1 in 20 women aged 30–49 years consult their general practitioner each year for HMB, which is the second highest-ranked reason for a hospital referral and accounts for 12% of all gynecologic referrals [4]. Overall, 8–38.9% of women experience HMB based on subjective assessments [5–7], and this condition has been linked to a lower quality of life and increased health care expenses [2, 8]. There are a number of factors that are known to be associated with HMB [6]; however, many of these factors are not fully understood.

Among Chinese population, research on HMB has been very limited. Up to now, there is only one study that has examined self-reported HMB, giving a prevalence of 36% in 2012 [9], no published study has addressed risk factors for HMB or the disturbances of women’s daily lives caused by the bleeding. In a community-based sample of Chinese women aged 18 to 50 years old living in Beijing, China, we aimed to: 1) investigate the prevalence of self-reported HMB and associated risk factors; and 2) evaluate the impact of menstrual bleeding on their daily lives in relation to HMB.

## Methods

### Study setting

A cross-sectional survey was conducted between October 2014 and July 2015 in Beijing, China. According to the 2010 Beijing population census data, occupations in women were classified into six major categories: 1) producers in agriculture, forestry, animal husbandry, fishery, and water conservancy; 2) business service personnel; 3) production, transport equipment operators, and related workers; 4) technical personnel; 5) clerk and related workers; and 6) students and others [10]. Therefore, this study extracted six corresponding occupational groups (farmer, business service personnel, worker, technical personnel, clerk, and student) to represent the entire study population.

### Participant eligibility and recruitment

Participation was restricted to women aged between 18 and 50 years of age, with current menstruation, and who had been living in Beijing for at least 6 months.

We calculated a minimum sample size of 2245 participants based on the assumption of 30% prevalence of HMB with a precision of 3% and a survey design effect of 2.58 to account for the interclass correlation of a cluster sampling design (see Additional file 1) [11]. The number was increased by 20% and considered expected losses. Therefore, the ultimate anticipatory sample size was determined to be 2800. We then proportionally allocated 2800 to each occupational group according to its percentage of the total population.

Within each occupation group, we used a cluster sampling design that included three steps. For example, in taking a sample selection of students, 1) schools were selected as “investigation units” by convenience sampling hosted by a committee of experts; 2) classes were defined as “clusters” and randomly selected in each investigation unit; and 3) individuals were selected randomly in each cluster. Steps 2 and 3 were repeated until a sufficient sample size was reached. Randomization was done with random number generator software. For farmers, investigation units and clusters were townships and village committees, respectively, whereas in the remaining occupation groups, companies and trade union committees were regarded as investigation units and clusters, respectively. Written informed consent was obtained from all participants before inclusion. The participation is voluntary, anonymous, and not reimbursed. The Peking University Institutional Review Board approved the study.

### Procedure

The questionnaire used in the current study was adapted specifically for Chinese women of reproductive age from the Menstrual Disorder of Teenagers (MDOT) Study questionnaire which showed sufficient reliability and validity [12]. The content of the questionnaire were reviewed by a group of gynecologists and public health experts, and a good validity was concluded. A pilot survey of 100 women was performed to further improve the overall layout and readability of the questionnaire base on their feedback. Eligible participants were given a 39-item questionnaire, which focused on the following topics: demographic characteristics, lifestyle factors, reproductive history, present clinically diagnosed diseases, self-perceived MBL, and interference of menstruation with daily life.

### Measures

#### Outcome

It has become increasingly apparent that whether menstrual bleeding causes problems should be determined by the woman herself, not by measuring MBL. In real clinical practice, women are encouraged to seek medical care if they feel that their menstrual cycle or amount of blood loss does not fall within the normal ranges [1]. Therefore, in the present study, HMB was identified based on self-assessment by the question asking whether participants experienced their menstrual bleeding as heavy, normal, light or no menstruation over the previous 3 months and, if no menstruation was experienced, the reason should be given.

#### Predictors

Body mass index (BMI) was calculated from self-reported height and weight data; according to the criteria for Chinese adults [13], participants with BMI < 18.5 kg/m^2^, BMI ≥ 18.5 kg/m^2^, BMI ≥ 24 kg/m^2^, and BMI ≥ 28 kg/m^2^ were diagnosed as underweight, normal, overweight, and obese, respectively. Smoking status was divided into four categories: non-smoker, occasional, regular (at least one cigarette per day for at least six consecutive months), and former smoker (quitting smoking for at least 6 months prior to the survey). Similarly, alcohol consumption was categorized as non-drinker, occasional (drinking alcohol less than one time per week), regular (drinking alcohol at least one time per week), and former drinker (quitting drinking for at least 6 months prior to the survey). Participants were also asked to report whether they have relevant diseases (including uterine pathology, coagulation disorders, thyroid disorders, and iron-deficiency anemia) confirmed by a physician. In China, diagnostic criteria for iron-deficiency anemia includes: 1) anemia (hemoglobin< 110 g/L in adult women); 2) microcytic: MCV < 80 fl, MCH < 27 pg, MCHC< 32%; and 3) iron deficiency: serum ferritin< 12 μg/L, increased total iron-binding capacity, decreased serum iron, decreased transferrin saturation [14].

#### Impact

The practical impact of bleeding was estimated by asking whether participants changed pads during nights or bled through pads over the previous 3 months; the possible answers were “yes” or “no”. Participants were also asked to rate how often they planned or refrained from activities (such as sports, parties, etc.) with regard to menstrual bleeding in the past 12 months, classified as every month, quite often, sometimes, and never; high interference was defined as answers including “every month” and “quite often”. Specific activities listed in the interference section were sport and exercise, holidays, choosing clothes, doing housework, social activities, sexual activity, and work/school performance. High interference was deemed to be present when participants answered “greatly affected” or “partly affected” to questions about menstrual impact on these activities in the past 12 months. The menstrual interference with work or school attendance was measured according to how many days participants were absent from work or school because of menstrual bleeding during the past 12 months, choosing from never, 1–5 days, 6–10 days, and > 10 days.

### Statistical analysis

Prevalence of HMB was calculated and then stratified by demographic, lifestyle, and reproductive characteristics. The associations between the presence of HMB and various characteristics, present diseases, and menstrual interference with daily life were evaluated using the Pearson chi-square or chi-square for trend test. We used logistic regression model to calculate unadjusted and adjusted odds ratios (ORs) with 95% confidence intervals (CIs) for HMB according to risk factors. All *P* values were 2-sided and were considered statistically significant if they were less than 0.05. Statistical analyses were performed using SPSS version 16 (IBM, Armonk, NY, USA).

## Results

There were a total of 2864 women selected and invited for the survey. Of these women, 2455 replied and returned the questionnaires, giving a response rate of 85.7%. 64 questionnaires were excluded because of missing information regarding self-perceived MBL. 35 participants had no menstruation in the past 3 months due to pregnancy/breastfeeding (*n* = 14), contraceptives (*n* = 16), operation (*n* = 4), and post-menopause (n = 1), leaving 2356 for final analyses. All participants were aged 18 to 50 years, with an average of 31.82 ± 8.73 years.

### Prevalence of self-reported HMB

Overall, the prevalence of women experiencing HMB was 18.2% (*n* = 429/2356). Table 1 presents the prevalence of HMB by age, occupation, BMI, lifestyle factors, reproductive history, and present clinically diagnosed diseases, which also shows the distributions of participants in terms of these characteristics. Prevalence of HMB increased with alcohol consumption and was highest in women who had three or more abortions. In addition, women with self-report uterine fibroids and iron-deficiency anemia were more likely to suffer from HMB than those without.

**Table 1:** Prevalence and odds ratio of HMB according to demographics, lifestyle factors, reproductive history, and present clinically diagnosed diseases

Characteristics	Participant	Participant experiencing HMB			
	N (%) ^a^	n	Prevalence (%)	*P* value	Crude OR (95%CI) ^d^
Overall	2356 (100.0)	429	18.2	–	–
Age (years)					
≤ 24	559 (23.7)	113	20.2	0.155 ^b^	1.24 (0.83–1.84)
25–29	511 (21.7)	93	18.2		1.09 (0.72–1.63)
30–34	461 (19.6)	79	17.1		1.01 (0.66–1.53)
35–39	331 (14.1)	66	19.9		1.21 (0.79–1.87)
40–44	259 (11.0)	38	14.7		0.84 (0.52–1.36)
≥ 45	235 (10.0)	40	17.0		Reference
Occupation ^e^					
Farmer	176 (7.5)	25	14.2	0.720 ^c^	Reference
Technical personnel	437 (18.5)	71	16.2		1.17 (0.72–1.92)
Business service personnel	834 (35.4)	158	18.9		1.41 (0.89–2.23)
Student	312 (13.2)	59	18.9		1.41 (0.85–2.34)
Worker	278 (11.8)	53	19.1		1.42 (0.85–2.39)
Clerk	319 (13.5)	63	19.7		1.49 (0.90–2.46)
BMI (kg/m^2^)					
18.5–23.9	1486 (63.1)	269	18.1	0.735 ^b^	Reference
< 18.5	303 (12.9)	64	21.1		1.21 (0.89–1.65)
24.0–27.9	419 (17.8)	69	16.5		0.89 (0.67–1.19)
≥ 28.0	99 (4.2)	18	18.2		1.01 (0.59–1.70)
Smoking					
Never	2235 (94.9)	406	18.2	0.869 ^c^	Reference
Occasionally	80 (3.4)	13	16.3		0.87 (0.48–1.60)
Regularly	26 (1.1)	6	23.1		1.35 (0.54–3.39)
Former smoker	9 (0.4)	2	22.2		1.29 (0.27–6.22)
Alcohol consumption					
Never	1287 (54.6)	199	15.5	< 0.001 ^c^	Reference
Occasionally	1021 (43.3)	219	21.5		1.49 (1.21–1.85)
Regularly	29 (1.2)	10	34.5		2.88 (1.32–6.28)
Former user	14 (0.6)	1	7.1		0.42 (0.06–3.23)
Number of pregnancies					
0	955 (40.5)	175	18.3	0.392 ^b^	Reference
1	508 (21.6)	81	15.9		0.85 (0.63–1.13)
2	423 (18.0)	72	17.0		0.91 (0.68–1.24)
3	208 (8.8)	41	19.7		1.09 (0.75–1.60)
≥ 4	73 (3.1)	19	26.0		1.57 (0.91–2.71)
Number of abortions					
0	1543 (65.5)	265	17.2	0.004 ^b^	Reference
1	400 (17.0)	71	17.8		1.04 (0.78–1.39)
2	178 (7.6)	38	21.4		1.31 (0.89–1.92)
≥ 3	53 (2.3)	19	35.9		2.70 (1.51–4.80)
Number of childbirths					
0	1034 (43.9)	198	19.2	0.076 ^b^	Reference
1	961 (40.8)	169	17.6		0.90 (0.72–1.13)
≥ 2	197 (8.4)	27	13.7		0.67 (0.43–1.04)
Uterine fibroids					
Yes	186 (7.9)	51	27.4	0.001 ^c^	1.79 (1.27–2.52)
No	2170 (92.1)	378	17.4		Reference
Coagulation disorders					
Yes	2 (0.1)	1	50.0	0.331 ^c^	4.50 (0.28–72.09)
No	2354 (99.9)	428	18.2		Reference
Thyroid disorders					
Yes	32 (1.4)	7	21.9	0.588 ^c^	1.26 (0.54–2.94)
No	2324 (98.6)	422	18.2		Reference
Iron-deficiency anemia					
Yes	151 (6.4)	38	25.2	0.022 ^c^	1.56 (1.06–2.29)
No	2205 (93.6)	391	17.7		Reference

*BMI* body mass index (calculated as weight in kilograms divided by the square of height in meters), *CI* confidence interval, *HMB* heavy menstrual bleeding, *OR* odds ratio

^a^Numbers or percentages might not add to total or 100 because of missing data

^b^Chi-square for trend test

^c^Pearson chi-square test

^d^OR calculated in univariate logistic regression analyses

^e^Occupations were classified into six groups according to the 2010 Beijing population census data: 1) producers in agriculture, forestry, animal husbandry, fishery, and water conservancy (= “farmer”); 2) business service personnel; 3) production, transport equipment operators, and related workers (= “worker”); 4) technical personnel; 5) clerk and related workers (= “clerk”); and 6) students

### Risk factors for HMB

Risk factors that were significantly and independently associated with the presence of HMB were uterine fibroids, alcohol drinking, and younger age (Table 2); additionally, multiple abortions (≥3) was associated with a more than two-fold increase in the risk of HMB compared with no prior abortion.

**Table 2:** Multivariate analysis of factors associated with the presence of HMB

Factors	Adjusted OR ^a^	95% CI	*P* value
Uterine fibroids			
Yes	2.12	1.42–3.16	< 0.001
No	Reference		
Age (years)			
≤ 24	1.86	1.13–3.08	0.015
25–29	1.79	1.09–2.94	0.022
30–34	1.58	0.97–2.58	0.067
35–39	1.75	1.07–2.89	0.027
40–44	1.00	0.58–1.73	0.989
≥ 45	Reference		
Alcohol consumption			
Never	Reference		
Occasionally	1.37	1.09–1.73	0.007
Regularly	2.78	1.20–6.46	0.017
Former user	0.51	0.06–4.01	0.519
Number of abortions			
0	Reference		
1	1.16	0.85–1.60	0.357
2	1.49	0.97–2.27	0.066
≥ 3	3.44	1.82–6.49	< 0.001

*CI* confidence interval, *HMB* heavy menstrual bleeding, *OR* odds ratio

^a^OR calculated in multivariate logistic regression analyses with a backward elimination method based on maximum likelihood

### Impact of HMB on women’s daily life

Women experiencing HMB felt more practical discomforts than those without HMB in terms of bleeding through pads (*n* = 225/429 [52.4%] versus *n* = 678/1927 [35.2%], *P* < 0.001) and changing pads during the night (*n* = 70/429 [16.3%] versus *n* = 112/1927 [5.8%], *P* < 0.001). Generally, they planned activities considering periods to a greater extent, kept themselves from more activities due to the menstrual bleeding, and were more frequently absent from work or school because of the bleeding (Table 3). Significant associations were found between the presence of HMB and high menstrual interference with all specific activities, apart from sexual activity, which was affected similarly between women with and without HMB (Table 3). However, only 18.9% (*n* = 81) of women experiencing HMB stated that they had sought health care for their heavy periods.

**Table 3:** Association between HMB and menstrual interference with daily life

	With HMB (*N* = 429)		Without HMB (*N* = 1927)		*P* value ^c^
	n	%	n	%	
In general ^a^					
Planning activities	86	20.1	288	15.0	0.009
Refraining from activities	55	12.8	136	7.1	< 0.001
Specific activity ^b^					
Sport and exercise	232	54.1	918	47.6	0.016
Holidays	195	45.5	771	40.0	0.038
Choosing clothes	192	44.8	699	36.3	0.001
Doing housework	148	34.5	567	29.4	0.039
Social activities	134	31.2	494	25.6	0.018
Sexual activity	122	28.4	554	28.8	0.886
Work/school performance	117	27.3	435	22.6	0.038
Absence from work/school					
Never	360	83.9	1727	89.6	0.004
1–5 days a year	61	14.2	175	9.1	
6–10 days a year	6	1.4	23	1.2	
> 10 days a year	2	0.5	2	0.1	

*HMB* heavy menstrual bleeding

^a^Number of participants reported high interference, which was defined as answers including “every month” and “quite often” to questions about how often they planned or refrained from activities regarding the menstrual bleeding in the past 12 months

^b^Number of participants reported high interference, which was defined as answers including “greatly affected” and “partly affected” to questions about menstrual interference with specific activities in the past 12 months

^c^Pearson chi-square test

## Discussion

The present study is the first one that entailed a community-based survey to address the effect of HMB on a wider population of Chinese women and associated risk factors. The results of our study showed that the prevalence of experienced HMB among Chinese women aged 18–50 years living in Beijing was 18.2%, which was consistent with the self-reported prevalence of 8–27% in other developing countries [5], but notably lower than the prevalence of 36% reported by Zhao et al. based on subjective assessments from 1021 Chinese women of reproductive age [9]. However, the study by Zhao et al. was conducted in a selected group of patients who attended medical centers for insertion of levonorgestrel-releasing intrauterine system and thus might lead to an overestimate of the HMB rate [9].

While HMB may occur in the presence of uterine pathology and other disorders, a broader view of this condition has shown that its potential risk factors also include age, lifestyle factors, and reproductive history [15]. Therefore, we performed a detailed evaluation of these factors for self-reported HMB, and found that the presence of uterine fibroids, increased alcohol consumption, younger age, and multiple abortions (≥3) were predictors of heavy bleeding.

Uterine fibroids are age-related and one of the most common gynecologic abnormalities. The association between the site, size, and number of fibroids and the amount of MBL has been well-recognized [16]. In the present study, the self-reported rate of uterine fibroids was 11.9% (*n* = 51/429) in HMB group, which is in accordance with previous studies [17, 18]. Accordingly, after adjusting for age and other predictors in the model, our findings also showed that the presence of uterine fibroids doubled the risk of an individual woman experiencing HMB.

In our study, the prevalence of heavy bleeding increased monotonically with a higher drinking level. A similar result was obtained from the Danish web-based pregnancy planning study, which linked high alcohol consumption (≥14 drinks per week) with a 48% increase in risk of heavy periods [19]. No association was found in another study conducted in Brazil [7]; however, our results are difficult to compare with the Brazilian study because the latter only assessed dichotomous levels of alcohol consumption (any consumption versus none). Recent evidence indicates that alcohol intake may elevate levels of testosterone, estradiol, and luteinizing hormone in premenopausal women [20, 21]. In turn, such hormonal imbalance may increase menstrual bleeding, which suggests biological plausibility for the association between alcohol drinking and heavy bleeding.

Previous research has suggested that younger women (< 26 years old) are significantly more likely than older women (> 37 years old) to regard moderate blood loss as very heavy [2]. Based on our findings, women aged ≤24 and 25–29 years had increased odds for reporting heavy periods compared with those aged 45–50 years. This finding is probably because older women are getting more accustomed to coping with menstrual bleeding and its consequences; the bleeding thus might have a greater negative impact on younger women, which makes the blood loss feel heavier. An inverse relationship between age and HMB was also observed in a recent Danish study of women aged 18–40 years [19].

Our results showed that having multiple abortions (≥3) was associated with an increased risk of HMB, while no incremental risk was found among women with fewer numbers of abortions. To the best of our knowledge, only a few studies have investigated whether women’s reproductive histories affected their perception of menstrual bleeding. For example, Santos et al. found no difference in the prevalence of self-reported HMB between women with and without a history of abortion [7]. However, women in that study were crudely classified into two categories (≥1 abortions versus none), making it difficult to detect a correlation between number of abortions and HMB. When women reporting more than one abortion in this study were combined into one group, there is no statistical difference in HMB prevalence between women with abortion history and those who have never had an abortion (*P* = 0.087).

HMB and multiple abortions (≥3) may have shared common etiologies, such as underlying bleeding disorders [22], which can partly explain the observed correlation between these two conditions. Estimates suggested that 20–29% of women with HMB had an underlying bleeding disorder [22, 23]; however, recognition is difficult as women often ignore their symptoms until serious bleeding events occur. Correspondingly, only one woman who reported HMB and had a clinically diagnosed coagulation disorder was identified in our survey. As our study did not distinguish miscarriages from induced abortions, ongoing studies with the screening of bleeding disorders and comprehensive reproductive histories are still needed to better understand and confirm this finding.

Obesity is an independent risk factor for several hormonal abnormalities, such as increased concentrations of testosterone and insulin, and reduced concentrations of the sex hormone-binding globulin [24], which inevitably influences the menstrual cycle. Along with the well-documented increased risk of menstrual irregularity, a limited number of studies reported a higher prevalence of heavy periods among obese women [7, 19, 25]. We also observed a slightly higher prevalence of HMB among obese women (18.2%) compared with normal (18.1%) or overweight women (16.5%); however, these results failed to reach statistical significance (*P* = 0.735), most likely due to a small sample size of the obese group (*n* = 99 [4.2%]).

To our knowledge, this study is also the first one to examine the perceived impact of HMB on women’s daily lives in a Chinese population. We noted that heavy bleeding was associated with more practical discomforts and limited family, professional, and social activities, which are supported by findings in other study population [2, 26]. There is evidence that the main impacts of HMB were physical and social issues, which also served as the main reasons why women consulted for their heavy periods [15]. However, based on our findings, a considerable high proportion of the women surveyed treated their HMB with indifference and appeared reluctant to seek gynecological consultation even when faced with social concerns arising from the bleeding. One reason that keeps women from seeking assistance is that they view heavy periods as good fertility or feminine [2], often accompanied by a belief that the menstrual bleeding, even if heavy, is a salubrious cleansing of their bodies [6, 27]. An alternative explanation is the traditional “culture of silence surrounding menstruation”, which restrains women from knowing how their own experiences compare with those of others [6]. Previous qualitative studies have shown that more information was required for women in terms of accessing health care services [15], indicating an opportunity for health education programs on menstruation.

Noticeably, the prevalence of self-reported iron-deficiency anemia in this sample is only 6.4%, which is markedly lower than the national rate of 10.9% amongst Chinese adult women based on laboratory results [28, 29]. Even among those who reported HMB, only 8.9% were self-reported as anemic. These data suggest that a significant percentage of women may have anemia that remains undiagnosed and untreated. Anemia resulting from iron deficiency adversely affects cognitive and motor development, causes fatigue and low productivity, and if left untreated, can produce severe symptoms and result in major medical complications [29]. Also, studies have shown that the correction of iron deficiency and anemia among women experiencing HMB significantly improves their health-related quality of life [30]. Therefore, women should be educated on the consequences of HMB and counseled to seek care when they perceive HMB. More importantly, clinician should be aware that the evaluation for iron deficiency and anemia is essentially important for women who present with HMB.

Strengths of this study include its community-based design, inclusion of a wide range of potential risk factors, and updated definition of HMB. This study also provided detailed information on the impact of heavy periods on daily life, which is necessary to piece together a complete picture of the burden of this condition in Chinese women. Our study also has limitations. First of all, because of cultural problems, women might feel uncomfortable discussing subject with their menstruation; however, they are more likely to be willing to be approached, with their schools, trade union committees or village committees engaging in organizing the survey, and this is the primary why a cluster sampling method with stratification by occupation was used in the present study. Although that the unemployed ones were excluded seems to be a limitation, unemployed females only accounted for an insignificant minority (1.2%) of the total women population aged 18–50 years living in Beijing in 2013 [31]. Second, the present data were all based on self-reports and might thus result in misclassification; however, it is virtually impossible to fully circumvent this limitation in survey studies due to the secretive nature of menstruation. Our assessment regarding HMB is reliable because it reflects women’s self-experience and shows a good correlation with both clinically relevant conditions like anemia and impacts on quality of life in previous studies [2, 32]. Finally, the cross-sectional design is a poor way to ascertain risk factors; further longitudinal studies are necessary to better understand the roles of these possible risk factors in causality and the effect of modifying them.

## Conclusions

In conclusion, HMB was a highly prevalent gynecological symptom among women of reproductive age living in Beijing, China. Uterine fibroids, multiple abortions (≥3), younger age and increased alcohol drinking were associated with the presence of HMB. Women experiencing HMB suffered from greater menstrual interference with daily lives while the percentage of health care seeking was unacceptably low. These factors highlight the need for information and health education programs that help raise awareness of the consequences of HMB such as iron deficiency and anemia, encourage women to seek assistance and thus improve their life quality.

## Additional file

Additional file 1: Sample size calculation. (PDF 146 kb)

## Abbreviations

BMI: Body mass index
CI: Confidence interval
HMB: Heavy menstrual bleeding
MBL: Menstrual blood loss
MDOT Study: The Menstrual Disorder of Teenagers Study
OR: Odds ratio
