# Supplementary material for: Heavy menstrual bleeding among women aged 18–50 years living in Beijing, China: prevalence, risk factors, and impact on daily life
Source: BMC Womens Health. 2019 Feb 4;19:27. doi: 10.1186/s12905-019-0726-1 (PMC6360654; doi:10.1186/s12905-019-0726-1)
Supplement: Supplementary file 1 — Sample size calculation. (PDF 146 kb) [file 12905_2019_726_MOESM1_ESM.pdf]

Additional file 1: Sample size calculation [1]

We calculated a minimum sample size of 2245 participants based on assumptions of 30% prevalence of HMB with a precision of 3% and a survey design effect of 2.58 to account for the interclass correlation of a cluster sampling design. Finally, the number was increased by 20% considering expected losses. Therefore, the ultimate anticipatory sample size was determined to be 2800.

We adopted stratified, cluster sampling strategy. For the start, we calculated a sample size under the assumption of simple random sampling in infinite population, using parameters and formula as presented below:

- P: The expected prevalence of menstrual bleeding. 30% was used according to previous studies;
- d: Absolute precision. Here, we took 3.0%;
- $1-\alpha$: Confidence level. We set 1-α = 95%, and $\mu_{\propto}$=1.96.

The sample size calculated by PASS software is 870 (n_1).

Then to correct for the cluster sampling design, n_1 was multiplied by the design effect (deff). The parameters and formula were presented below:

- m: The average sample size of clusters. We assumed the average size of the clusters was 80;
- roh: Interclass correlation coefficient. Here, we took 0.02 for the study group.

$$\mathrm{deff} = 1 + (\mathrm{m} - 1) \times \mathrm{roh} = 1 + (80 - 1) \times 0.02 = 2.58$$

$$n_2 = deff \times n_1 = 870 \times 2.58 = 2245$$

Finally, the number was increased by 20% considering expected losses.

$$n_3 = n_2 \times (1+20\%) = 2245 \times 1.2 = 2694$$

Therefore, the ultimate anticipatory sample size was determined to be 2800.

Reference

1. Chopra A, Rao NC, Gupta N, et al. Influence of behavioral determinants on deviation of body mass index among 12-15 years old school children of Panchkula. Epidemiol Health. 2014; 36:e2014021
